# Parent–Child Cohesion, Basic Psychological Needs Satisfaction, and Emotional Adaptation in Left-Behind Children in China: An Indirect Effects Model

**DOI:** 10.3389/fpsyg.2018.01023

**Published:** 2018-06-21

**Authors:** Jingjin Shao, Lei Zhang, Yining Ren, Luxia Xiao, Qinghua Zhang

**Affiliations:** ^1^Centre of Mental Health Education, Faculty of Psychology, Southwest University, Beibei, China; ^2^Faculty of Psychology, Southwest University, Beibei, China; ^3^Department of Tourism and Art for Humanity, Chongqing Youth Vocational & Technical College, Chongqing, China

**Keywords:** left-behind children, parent–child cohesion, emotional adaptation, psychological needs satisfaction, gender difference

## Abstract

This study aimed to validate an indirect effects model of parent–child cohesion in emotional adaptation (i.e., loneliness and depression) via basic psychological needs satisfaction in Chinese left-behind children as well as the applicability of the model to both genders. A cross-sectional study was conducted and included 1,250 children aged between 9 and 12 years (635 left-behind children and 615 non-left-behind children) from rural primary schools. The results showed that: (1) relative to non-left-behind children, left-behind children exhibited significantly higher loneliness and depression scores and greater disadvantages involving father–child cohesion, mother–child cohesion, and psychological needs satisfaction. (2) Father– and mother–child cohesion were significantly negatively correlated with loneliness and depression and significantly positively correlated with psychological needs satisfaction in left-behind children. (3) Through structural equation modeling showed that psychological needs satisfaction partially mediated the relationship between parent–child cohesion and emotional outcomes in left-behind children. (4) Through multi-group analyses showed significant gender differences in structural weighting between parent–child cohesion and emotional adaptation, in that parent–child cohesion in left-behind boys was a stronger negative predictor of unfavorable emotional outcomes relative to that observed in left-behind girls, while psychological needs satisfaction in left-behind girls was a stronger negative predictor of unfavorable emotional outcomes relative to that observed in left-behind boys. The implications of these findings for interventions directed at Chinese left-behind children were discussed.

## Introduction

With the acceleration of urbanization and the development of a market economy over the past 30 years, China has undergone one of the largest rural-to-urban migrations in human history. Moreover, because of the urban-rural dual economic structure, many parents have been forced to move to urban areas to seek employment and leave their children with extended family members, creating a vulnerable group of “left-behind children.” Tens of millions of left-behind children in rural areas have become part a unique disadvantaged group in a special historical period of China, living under the restrictions of urban “Hukou,” an official household residency permit system that grants access to social services including education, healthcare, and the right to buy homes. Left-behind children are generally younger than 17 years of age and remain at home in rural areas while one or both parents migrate to urban areas to work for at least 6 months (All-China Women’s Federation, 2013; Zhao et al., 2015). According to the latest statistics, there are currently approximately 9.02 million left-behind children in China (Ministry of Civil Affairs, 2016). The phenomenon of left-behind children, as a disadvantaged group resulting from China’s economic and social transition, is expected to last for a considerable period.

According to population characteristics, all left-behind children face the common issue of parental absence caused by parents’ long-term rural-to-urban migration, which destroys normal family structure and affection. The characteristics of this parental absence often generate considerable detrimental effect on left-behind children’s emotional adaptations (Cheng et al., 2010; Wen and Lin, 2012; Zhao et al., 2014; Cheng and Sun, 2015). Emotional adaptation typically refers to the unpleasant emotions or negative emotions experienced by children, such as loneliness, depression and anxiety (Fan, 2011; Cicchetti and Rogosch, 2012; Zhao et al., 2015). Relative to their age-matched peers, left-behind children, who lack family affection and connections, tend to report higher levels of loneliness, depression, anxiety, grievance, self-abasement, and deviant behavior (Hao and Cui, 2007; Liu et al., 2007; Fan et al., 2009; Liu et al., 2012). Several review articles have provided a clear demonstration of the relationship between parental rural-to-urban migration and unfavorable developmental outcomes across the emotional, social, and academic domains of functioning (Cheng and Sun, 2015; Shen et al., 2015; Wang and Mesman, 2015). In addition, the experience of being left behind during childhood could exert long-term detrimental effects on children’s emotional adjustment in adulthood, which is associated with high levels of depression, anxiety, loneliness, and low self-esteem (Li et al., 2009). However, left-behind children are not the same as problem children. Many left-behind children do not exhibit maladjustment in disadvantageous situations and some have achieved outstanding performance in certain fields (Wen and Lin, 2012; Zhao et al., 2013b). In this context, greater attention should be paid to resilience and protective factors in left-behind children.

Left-behind children’s healthy development is determined by their possession of characteristics that protect them against these risks or adverse environments. According to Bronfenbrenner’s ecological systems theory, numerous developmental psychologists have examined the contributions made by overlapping and interacting social contexts in child development (i.e., family members and peers) and the interacting with children’s intrapersonal characteristics, which shapes their developmental trajectories. Parent–child cohesion generally refers to intimate emotional connections between parents and children, which could be manifested by positive interaction and feelings of closeness between foster caregivers and children (Zhang et al., 2006). As an indicator of parent–child relationships, parent–child cohesion is generally viewed as the basis of children’s healthy development. Specifically, parent–child cohesion is conducive to healthy growth and serves as a protective factor, helping children to overcome risks encountered during development (Formoso et al., 2000; Bean et al., 2006; Zhao et al., 2013b). Numerous studies have demonstrated that parent–child cohesion can protect healthy children’s development (Bean et al., 2006) and reduce the deleterious effects of loneliness, depression, and problematic behavior in children (Formoso et al., 2000; Lamborn and Felbab, 2003; Kliewer et al., 2006). Steinberg (2007) emphasized that no other family factors are as important to children’s development as the quality of parent–child relationships.

Parents’ migration to urban areas for employment leads to fundamental alterations in parent–child interaction, which becomes characterized by long-term and -distance separation, lack of face-to-face interaction, and infrequent communication, and to some extent, parents become mere bystanders in the lives of their left-behind children (Li, 2002; Su et al., 2013). However, affectionate connections between parents and left-behind children are not severed entirely (Zhao et al., 2013b). Migrant parents maintain affection and bond with their children via telephone, messages, and the QQ or WeChat communication applications, and they return home during busy farming seasons. Research examining the role of parent–child cohesion in the development of left-behind children has followed two main aspects: (1) as a means of protecting emotional, psychological adaptation, and preventing deviant behavior in left-behind children (Chen et al., 2009; Zhao et al., 2013a,b; Zhao J. X. et al., 2016), and (2) the mechanism of parent–child cohesion and family atmosphere on emotional adaptation in left-behind children (Fan et al., 2013; Yang et al., 2014). These researches have provided preliminary evidence concerning the protection of parent–child cohesion in the development of left-behind children. However, research focusing on left-behind children during the middle stage of childhood, roughly between the ages of 9 and 12 years, is required. Parent–child relationships are considered pivotal resources in healthy child development (Pittman et al., 2011), and parents’ roles in their children’s development are not easily replaced by others. Previous research has suggested that the role played by parent–child relationships in physical and mental development could be more important during middle childhood than it is during adolescence (Collins and Russell, 1991; Feldman, 2006). For example, Liu et al. (2009) reported that children who were separated from their parents at a young age exhibited high levels of anxiety and depression. This phenomenon could occur because early experiences of closeness and cohesion with parents are important in the development of secure attachment relationships (Guo et al., 2015), while separation from and conflict with parents could lead to the development of insecure attachment relationships in left-behind children. However, the mechanisms via which parent–child cohesion influences emotional adaptation in left-behind children during middle childhood requires further exploration.

Protective factors of individual development include both environmental factors (i.e., family and parents) and a series of individual factors (Luthar et al., 2000; Zeng and Li, 2003). Of these factors, the satisfaction of basic psychological needs is considered to exert an important effect on the promotion of individuals’ emotional adaptation. According to self-determination theory (Ryan and Deci, 2000), humans have three fundamental, universal psychological needs: autonomy, competence, and relatedness. The need for autonomy refers to individuals’ need to make their own choices and respect their choices; the need for competence refers to individuals’ need to accomplish difficult activities or tasks in a competent manner; and the need for relatedness refers to individuals’ need for closeness, support, and connectedness to their environment or other people (Deci and Ryan, 2000; Ryan and Deci, 2000). These three needs are inherent and indispensable, because they serve as essential psychological “nutrients” for well-being and thriving. Individuals achieve positive development if their basic psychological needs are satisfied; otherwise, they encounter developmental problems or maladjustment (Deci and Ryan, 2008). From this perspective, satisfaction of individuals’ psychological needs could be an important mediator of the influences of social environmental factors (i.e., school and family) on individuals’ healthy development. The satisfaction of basic psychological needs is a pivotal motivational mechanism that facilitates interpretation of the impact of social environmental factors on child development. In other words, by satisfying basic psychological needs, social environmental factors encourage individuals to maintain positive development, to ensure healthy growth and favorable emotional and behavioral development. Previous research has reported that parent–child cohesion, which involves a family microenvironment that fulfills children’s basic psychological needs (Levin and Currie, 2010; Von Korff and Grotevant, 2011), may exert further effects on emotional adaptation (i.e., loneliness and depression) in left-behind children. Furthermore, Guo et al. (2014) studied parent–adolescent communication in rural areas and showed that it influenced adolescents’ well-being indirectly via autonomy, competence, and relatedness. In accordance with this theory, it is reasonable to hypothesize that parent–child cohesion could affect loneliness and depression both indirectly, via basic needs satisfaction, and directly. The primary aim of the study was to evaluate an indirect effects model of the role of parent–child cohesion in emotional adaptation via psychological needs satisfaction in Chinese left-behind children.

In addition, the study examined the applicability of the indirect model to left-behind children of both genders. Gender schema theory and previous research examining the psycho-sociological development of children suggested that the relationships between parent–child cohesion, basic psychological needs satisfaction, and unfavorable emotional outcomes could exhibit gender differences. Gender schemata refer to the psychological patterns of individuals’ different gender-determined expectations. For example, parents use different parenting styles for boys and girls because of their own expectations. Specifically, girls could be taught to well bred, gentle, and compliant, while boys are likely to be encouraged to be independent, self-confident, and strong. Parents’ gender role expectations could exert imperceptible influences on children’s gender role concepts, response style, and personality formation. Moreover, some research has shown that girls exhibited stronger relational orientation and emotional need, relative to that observed in boys (Stroud et al., 2002), and could be more sensitive to parent–child cohesion. Therefore, relative to boys, girls maintained better relationships with family members and were more likely to perceive parents’ concerns and less likely to perceive parental control. Furthermore, extant literature (Steinberg, 1990; Fuligni and Zhang, 2004; Zhang and Fuligni, 2006; Wang and Zhang, 2007) has demonstrated a significant gender difference in parent–adolescent relationships, in that adolescent girls enjoyed significantly stronger cohesion and affectionate connections with their mothers than adolescent boys did. In other words, adolescent girls’ relationships with their parents were closer and more harmonious relative to those observed in adolescent boys. However, Xiao and Xu (2009) posited that adverse family connections exerted much stronger detrimental effects on boys than they did on girls. Studies involving left-behind children have produced similar findings. For example, Zhao et al. (2013a) showed that left-behind girls maintained significantly closer relationships with their parents relative to those observed in boys. However, many previous studies often consider gender as a covariant (Zhao et al., 2013a; Zhao J. X. et al., 2016), whereas some studies examining basic psychological needs considered the interaction between gender and their study variables (Fang et al., 2012; Zhao K. et al., 2016). Therefore, the secondary aim of the study was to determine the applicability of the hypothesized indirect model to both genders using multi-group analyses.

### Purpose of the Study

Based on the comparison of parent–child cohesion, basic psychological needs, and emotional adaptation between rural left-behind children and non-left-behind children, the study sought to evaluate an indirect effects model of parent–child cohesion in emotional adaptation via basic psychological needs satisfaction, and determine the applicability of the model to both genders. In particular, our purpose was threefold: (a) to determine whether levels of depression and loneliness in left-behind children were significantly higher relative to those observed in non-left-behind children, while disadvantaged in parent–child cohesion and basic psychological needs satisfaction; (b) to determine whether parent–child cohesion influenced emotional adaptation via basic psychological needs satisfaction in left-behind children; and (c) to examine the moderating role of gender differences in the hypothesized indirect effects model using multi-group analyses. The findings should clarify the influence of the experience of being left behind on children’s development, identify the potential mechanism underlying the influence of parent–child cohesion on unfavorable emotional outcomes, and provide empirical evidence for future research involving emotional adaptation interventions for left-behind children in China.

## Materials and Methods

### Participants

Data were collected as part of the first wave of an ongoing longitudinal study (i.e., the Development of Left-Behind Children Project). In total, 1,287 participants were recruited from 12 elementary schools in rural areas in Shanxi, Guizhou, and Sichuan provinces in China, which have large populations of migrating laborers. Of the collected questionnaires, 33 were returned incomplete and 4 included multivariate outliers (± 3 standard deviations); therefore, they were excluded from the analyses. Missing data were imputed using predicted values based on multiple regressions including a set of sociodemographic variables, with regression coefficients estimated via bootstrapping. Ultimately, 1,250 valid questionnaires were collected, and the sample included 662 boys and 588 girls aged between 9 and 12 years, with a mean age of 11.22 ± 0.57 years. Participants were divided into three groups according to parental migration status (i.e., whether one parent, both parents, or neither parent had migrated to an urban area for employment): both-parents left-behind children with both parents migrated (*n* = 187, 15.0%); one-parent left-behind children with one parent migrated (*n* = 448, 35.8%); and non-left behind children (*n* = 615, 49.2%). Of the left-behind children, 337 (53.1%) were boys and 298 (46.9%) were girls.

### Procedure

The study protocol was approved by the institutional review board at the institution with which the first authors were affiliated. Prior to initiation of the study, all children and their parents or guardians were informed that they had the right to opt-out at any time and the Committee has approved the procedures. Data were collected by group-administered in participants’ classrooms at prearranged times. The experimenters included psychologists and graduate students with developmental psychology as an academic major at a large-scale university in south-west China. During the implementation of the study, the research administrators adhered strictly to a regulated program and read the instructions and interpreted response requirements for all participants. Participants completed the questionnaires individually and anonymously. The entire experiment lasted approximately 40 min, and participants received a small gift upon completion of the questionnaire.

### Measures

#### Parent–Child Cohesion

Participants’ perception of their cohesion with their parents was assessed using the Cohesion subscale of the Family Adaptation and Cohesion Evaluation Scales II (Olson et al., 1979). The subscale includes the same 10 items for mothers and fathers [e.g., “My mother/father and I feel very close to each other” and “My mother/father and I avoid each other at home (reverse coded)”]. Participants’ responses are provided using a five-point scale ranging from 1 (*almost never*) to 5 (*almost always*). Higher scores indicate higher levels of perceived parent–child cohesion involving fathers or mothers. The scale has been used widely in previous studies examining Chinese parent–adolescent cohesion. Cronbach’s α for father– and mother–child cohesion were 0.81 and 0.76, respectively (Zhang et al., 2006). In the current study, Cronbach’s α for father– and mother–child cohesion were 0.78 and 0.74, respectively.

#### Basic Needs Satisfaction

Satisfaction of participants’ basic psychological needs was assessed using the Basic Needs Satisfaction Scale – in General, which was developed by Deci and Ryan (2000). The Chinese version was validated in a previous study (Yu et al., 2012). It consists of three subscales pertaining to psychological needs in everyday life: Autonomy (seven items; e.g., “I feel like I am free to decide for myself how to live my life”), Competence (six items; e.g., “I have been able to learn interesting new skills recently”), and Relatedness (eight items; e.g., “I really like the people I interact with”). Participants’ responses are providing using a five-point scale ranging from 1 (*not at all true*) to 5 (*very true*). Higher scores indicate greater satisfaction of participants’ basic psychological needs. In the current study, Cronbach’s α for the Autonomy, Competence, and Relatedness was 0.64, 0.62, and 0.74, respectively.

#### Emotional Adaptation

Two indicators of emotional adaptation, loneliness and depression were assessed, respectively. Loneliness was assessed using the Chinese version of the Child Loneliness Scale (Asher et al., 1984). The scale consists of 24 items, with 16 items pertaining to loneliness (e.g., “I feel alone” and “I have nobody to talk to”) and eight filler items (e.g., “I like to read”). Participants’ responses are provided using a five-point scale ranging from 1(*not at true*) to 5 (*always true*). Mean scores are calculated for the 16 items pertaining to loneliness, and higher scores indicate greater loneliness. The scale has been used in previous studies (e.g., Tian et al., 2012; Zhao et al., 2015). Cronbach’s α for the scale was 0.83 in the current study.

Depression was assessed using the Chinese version of the Depression Self-Rating Scale for Children (Birleson, 1981). It is suitable for use with children aged 8–13 years and includes 18 items. Participants’ responses, which indicate the extent of their agreement with the items, are provided using a three-point scale ranging from 0 (*never*) to 2 (*mostly*). Higher scores indicate stronger likelihood of depression. The scale has demonstrated good reliability and validity for use with samples of Chinese children. Cronbach’s α of the scale was 0.73 (Su et al., 2003). In the current study, Cronbach’s α for the scale was 0.70.

### Data Analyses

Analyses of variance (ANOVAs) were performed to compare depression, loneliness, father–child cohesion, mother–child cohesion, autonomy, competence, and relatedness between groups, which were categorized according to parental migration status. Structural equation modeling was used to validate the hypothetical indirect effects model in the prediction of emotional adaptation. The following indices were used to evaluate overall model fit: χ^2^ goodness-of-fit statistic, goodness-of-fit index (GFI), adjusted GFI (AGFI), comparative fit index (CFI), and root mean square error of approximation (RMSEA). In addition, multi-group analysis, which involves a set of procedures developed to evaluate the invariance through structural equation modeling between groups, was used to perform probability testing of equivalence for the indirect effects model. All statistical analyses were preformed using SPSS and AMOS for Windows 22.0.

## Results

### Preliminary Analyses

Children in rural areas were divided into three groups based on categories used in previous research (Zhao et al., 2013b): both-parents left-behind children (*n* = 187), one-parent left-behind children (*n* = 448) and non-left-behind children (*n* = 615). Means, standard deviations, and ANOVA results for the main study variables are shown in **Table 1**.

**Table 1:** Mean differences in main study variables between groups.

	One parent migrated (*n* = 448) *M*± *SD*	Both parent migrated (*n* = 187) *M*± *SD*	Non-left-behind children (*n* = 615) *M*±*SD*	*F*	*p*	η^2^
Depression	30.42 ± 4.57	30.47 ± 4.45	29.47 ± 4.99	6.42^∗∗^	0.002	0.01
Loneliness	36.08 ± 10.43	35.06 ± 10.35	33.45 ± 10.80	8.15^∗∗∗^	0.000	0.02
Mother–child cohesion	24.44 ± 4.73	23.10 ± 5.40	25.42 ± 4.96	16.99^∗∗∗^	0.000	0.03
Father–child cohesion	24.07 ± 5.24	23.47 ± 5.39	25.06 ± 5.08	8.80^∗∗∗^	0.000	0.02
Autonomy	23.87 ± 4.48	23.53 ± 4.11	24.49 ± 4.75	4.26^∗^	0.014	0.01
Competence	20.92 ± 3.75	20.83 ± 4.84	21.47 ± 4.03	3.20^∗^	0.041	0.01
Relatedness	28.99 ± 6.02	29.24 ± 5.56	30.28 ± 6.02	6.64^∗∗^	0.001	0.01

*^∗^P < 0.05; ^∗∗^P < 0.01; ^∗∗∗^P < 0.001.*

The results showed that depression, loneliness, father–child cohesion, mother–child cohesion, autonomy, competence, and relatedness differed significantly between the three groups (**Table 1**). Specifically, relative to those observed for non-left behind children (i.e., comparison group), both groups of left-behind children exhibited significantly higher depression, *F* = 6.42, *p* < 0.01, η^2^ = 0.01, and loneliness, *F* = 8.15, *p* < 0.001, *^2^* = 0.02, scores and significantly lower mother–child cohesion, *F* = 16.99, *p* < 0.001, η^2^ = 0.03, father–child cohesion, *F* = 8.80, *p* < 0.001, η^2^ = 0.02, Autonomy, *F* = 4.26, *p* < 0.05, η^2^ = 0.01, Competence, *F* = 3.20, *p* < 0.05, η^2^ = 0.01, and Relatedness, *F* = 6.64, *p* < 0.01, η^2^ = 0.01, scores. However, no significant differences in these variables were observed between the two groups of left-behind children.

Pearson’s correlation coefficients for associations between mother–child cohesion, father–child cohesion, autonomy, competence, relatedness, depression, and loneliness in left-behind children are presented in **Table 2**. The results showed that mother– and father–child cohesion were negatively correlated with loneliness and depression and positively correlated with autonomy, competence, and relatedness in left-behind children. In addition, autonomy, competence, and relatedness were significantly negatively correlated with loneliness and depression in left-behind children.

**Table 2:** Correlations between study variables for left-behind children (*n* = 635).

	1	2	3	4	5	6	7
(1) Mother–child cohesion	1						
(2) Father–child cohesion	0.57**	1					
(3) Autonomy	0.38**	0.38**	1				
(4) Competence	0.31**	0.31**	0.47**	1			
(5) Relatedness	0.44**	0.43**	0.56**	0.48**	1		
(6) Depression	–0.42**	–0.42**	–0.39**	–0.38**	–0.46**	1	
(7) Loneliness	–0.40**	–0.35**	–0.40**	–0.38**	–0.56**	0.50**	1

*^∗^*P* < 0.05; ^∗∗^*P* < 0.01; ^∗∗∗^*P* < 0.001.*

### Validation of the Indirect Effects Model

The precondition for validation of the indirect effects model was that parent–child cohesion predicted unfavorable emotional adaptation. Based on the results of the Pearson’s correlation analysis, a latent variable model was created, with parent–child cohesion, including father– and mother–child cohesion, included as the exogenous latent variable and the independent variable. Depression and loneliness, which served as the two indicators for a latent emotional adaptation variable, were included in our structural model. The fit indices for the model were satisfactory: χ^2^ = 1.055, *df* = 1; GFI = 0.999; AGFI = 0.992; CFI = 1.000; RMSEA = 0.009. Parent–child cohesion exerted a negative predictive effect on emotional adaptation in left-behind children, β = -0.79, *t* = -10.06, *p* < 0.001).

**FIGURE 1:**
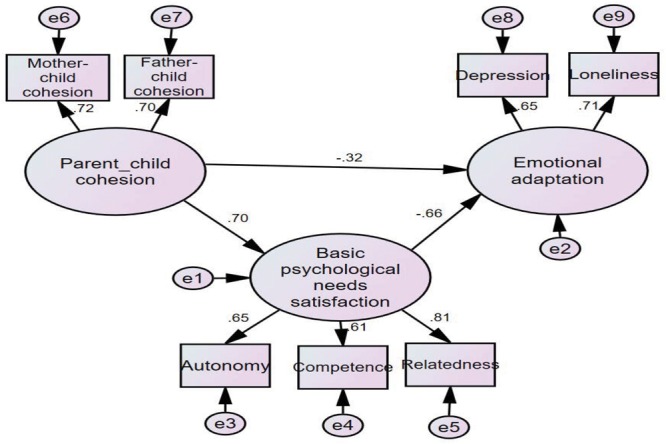
Indirect effects model. Note: Only standardized regression coefficients are used in the figure, and *P*-values are all <0.05.

In accordance with Wen et al. (2004), the indirect mediating effect model of basic psychological needs in left-behind children was verified using structural equation modeling including the total sample of left-behind children, with autonomy, competence, and relatedness as observed variables. The results indicated that basic psychological needs satisfaction partially mediated the effects of parent–child cohesion on emotional adaptation in left-behind children. The standard path coefficient for the model is shown in **Figure 1**. The fit indices for the model were as follows: χ^2^ = 20.83, *df* = 11; GFI = 0.991; AGFI = 0.976; CFI = 0.992; and RMSEA = 0.038.

According to the model analysis (**Figure 1**), parent–child cohesion influenced emotional adaptation both directly, β = -0.32, *t* = -10.06, *p* < 0.001, and indirectly via psychological needs satisfaction in left-behind children. The indirect effect explained 58.5% of the total variance in emotional adaptation, which indicated that basic psychological needs satisfaction partially mediated parent–child cohesion and emotional adaptation in left-behind children. Based on the Sobel test results regarding the mediating effect of basic psychological needs satisfaction, the indirect effect of basic psychological needs satisfaction on emotional adaptation was significant in left-behind children (*Z* = -6.21, *p* < 0.001).

### Multi-Group Analyses of the Indirect Effects Model According to Gender

**FIGURE 2:**
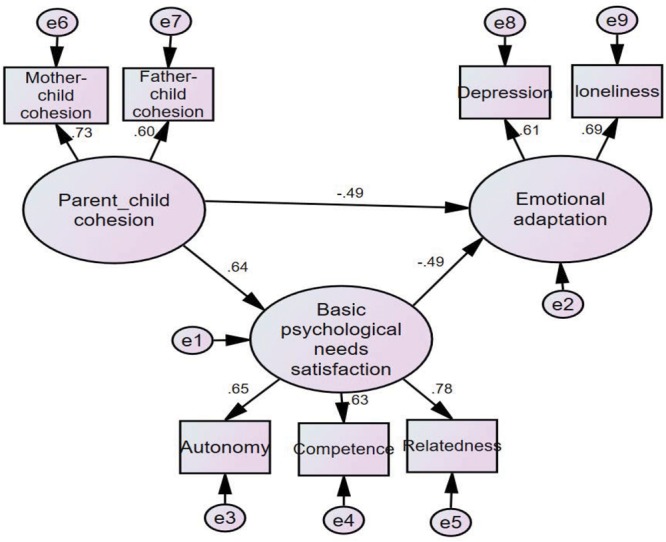
Indirect effects model (boys).

**FIGURE 3:**
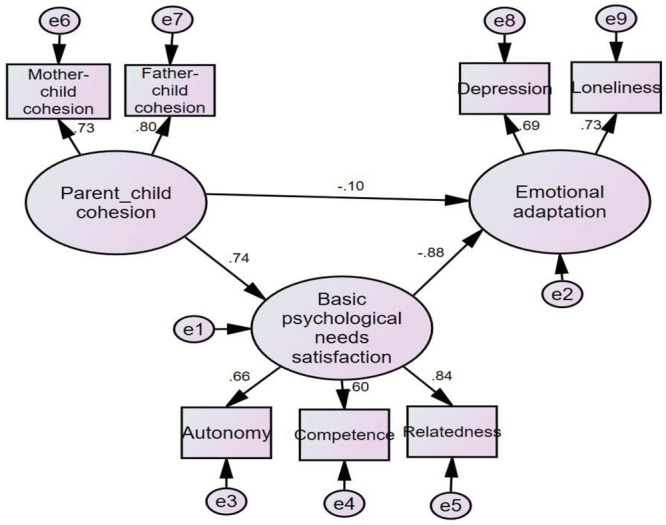
Indirect effects model (girls).

To examine the applicability of the indirect effects model with basic psychological needs satisfaction for both genders, using multi-group analysis of structural invariance, models were established for both boys and girls to allow free estimation. Good fit indices (**Table 3**) were observed for the indirect effects models for both boys (**Figure 2**) and girls (**Figure 3**), demonstrating the feasibility of multi-group comparison in structural equation modeling to the above indirect effects model.

**Table 3:** Comparison of indirect effects models.

Model	χ^2^	*df*	χ^2^*/df*	GFI	AGFI	CFI	RMSEA	*Δ*χ^2^ *(Δdf)*	*p*
M_boys_	11.441	11	1.040	0.990	0.976	0.999	0.011	–	
M_girls_	11.650	11	1.059	0.989	0.972	0.999	0.014	–	
M_1 Unconstrained_	23.092	22	1.050	0.990	0.974	0.999	0.009	–	
M_2 Measurementweights_	27.905	26	1.073	0.988	0.973	0.999	0.011	4.813 (4)	0.370
M_3 Structuralweights_	36.209	29	1.249	0.984	0.984	0.994	0.020	8.304 (3)	0.040

*Abbreviations: GFI, goodness-of-fit index; AGFI, adjusted goodness-of-fit index; CFI, comparative fit index; RMSEA, root mean square error of approximation*.

Thereafter, two types of sample (i.e., boys and girls) were generated for multi-group comparison in the structural equation modeling. The fit indices are presented in **Table 3**. Specifically, compared to the baseline model (Model 1), the additional constraints imposed in the measurement model (Model 2) did not result in a deterioration, *Δ*χ^2^= 4.81, *Δdf* = 4, *P*> 0.05, indicating that the measurement weights were invariant between boys and girls. However, compared to Model 2, the additional constraints imposed in the structural model (Model 3) led to a deterioration, *Δ*χ^2^= 8.30, *Δdf* = 3, *P* < 0.05. It appears that there are some significant differences in the indirect effects of overall parent–child cohesion on emotional adaptation between boys and girls.

Subsequent multi-group analyses were performed with gender group pairing to identify between-group differences. We examined the critical ratios (CRs) for differences in parameters between groups, with a *z*-score ≥ 1.96 considered significantly different. It showed that the effects of parent–child cohesion, CR = 2.24, *p* < 0.05, and basic psychological needs satisfaction, CR = -2.55, *p* < 0.05, on emotional adaptation in left-behind children exhibited significant gender differences. Parent–child cohesion was a significant positive predictor of basic psychological needs satisfaction; however, no significant gender differences were observed, boys: β = 0.64, *p* < 0.05; girls: β = 0.74, *p* < 0.05. The results verified that the indirect effects model of the effect of parent–child cohesion on emotional adaptation via basic psychological needs satisfaction is applicable to both boys and girls. However, there were significant gender differences in the structural weights for the two paths. Specifically, parent–child cohesion in left-behind boys exerted a significantly stronger negative predictive effect on emotional adaptation relative to that observed in left-behind girls, while psychological needs satisfaction in left-behind girls exerted a significantly stronger negative predictive effect on emotional adaptation relative to that observed in left-behind boys.

## Discussion

The results of the study showed that long-term parental absence due to rural-to-urban migration exerted adverse effects on development in Chinese left-behind children in middle childhood. Consistent with our hypotheses, the structural equation modeling indicated that parent–child cohesion exerted an indirect effect on emotional adaptation via basic psychological needs satisfaction in left-behind children, and this effect was moderated by gender. The findings not only support the resilience model of child development to some extent (Luthar et al., 2000) but also provide essential empirical evidence to enhance understanding of the important role of parents in the developmental process in Chinese left-behind children.

The results showed that left-behind children exhibited disadvantages in emotional adaptation as a consequence of their parents’ absence because of rural-to-urban migration. Left-behind children tend to report higher levels of perceived anxiety and depression relative to non-left-children peers, which is consistent with previous findings (Fan et al., 2009; Cheng and Sun, 2015; Shen et al., 2015; Wang and Mesman, 2015). Left-behind children’s father–child cohesion, mother–child cohesion, autonomy, relatedness, and competence scores were significantly lower relative to those observed in non-left-behind children who lived with their parents, which confirmed the findings of previous research. For instance, Lin and He (2012) reported that left-behind children experienced greater parent–child alienation relative to non-left-behind children. These results indicated that parents’ rural-to-urban migration for employment places children in disadvantageous situations, and their long-term absence results in sense of alienation. This absence of intimate emotional bonding could be related to lack of direct communication and contact between left-behind children and parents (Li, 2002; Su et al., 2013), because this type of affectionate connection, which occurs only through physical closeness, including physical contact, caresses, kisses, and clear demonstrations of affection, between parents and their children (Valtolina and Colombo, 2012). Moreover, left-behind children were in a worse position with respect to basic psychological needs satisfaction (i.e., autonomy, competence, and relatedness), relative to that of children living with their parents. This disadvantage appeared to result partly from immature development and high dependence on family and parents in left-behind children in middle childhood. Moreover, “lack of parental love” could increase children’s risk of emotional distress resulting from inadequate parental care, supervision, support, and guidance and impaired parent–child bonding (Wen and Lin, 2012; Zhao et al., 2015). In other words, left-behind children tend to be more vulnerable relative to non-left-behind children.

Through structural equation modeling showed that parent–child cohesion was a significant negative predictor of unfavorable emotional outcomes in left-behind children, indicating that it is a pivotal resource and exerts critical protective effects on healthy child development (Bean et al., 2006). Moreover, as an essential developmental resource (Pittman et al., 2011), it exerted protective effects on left-children’s emotional adaptation despite the long-term separation from their parents, which supports previous research findings (Kliewer et al., 2006; Zhao et al., 2013b, 2015). This effect could occur because left-behind children perceive parental love and concern directly, engage in intimate self-disclosure, and maintain connections to their parents because of high cohesion levels (Vieno et al., 2009). For example, they would confide in parents about difficulties and unhappy events at school and in life. Accordingly, migrant parents should communicate with their children regularly and help them to cope with difficulties and challenges.

In addition, basic psychological needs satisfaction (i.e., autonomy, competence, and relatedness) reduced unfavorable emotional outcomes in left-behind children, which is consistent with results of extant research (Wei et al., 2005). Guo et al. (2014) showed that basic psychological needs satisfaction predicted perceived well-being in rural children; this supports self-determination theory, which posits that psychological needs satisfaction serves as inherent psychological “nutrition” and plays a critical role in psychological development and integration. In contrast, threats or reductions in psychological needs satisfaction damage physical and psychological development (Ryan and Deci, 2000). Many studies have confirmed that basic psychological needs satisfaction predicted responses to pressure in life, social adaptation, and problematic behavior (Fang et al., 2012; Yu et al., 2012; Zhao K. et al., 2016). The current study was one of the first to examine the critical role of basic psychological needs satisfaction in the relationship between parent–child cohesion and emotional adaptation in Chinese left-behind children and showed that it played an important role in reducing negative outcomes. These results highlight the need to consider satisfaction of basic needs in the development of interventions to facilitate positive development in Chinese left-behind children facing adversity resulting from parents’ long-term rural-to-urban migration.

The results also showed that parent–child cohesion, as an environmental factor, could influence left-behind children’s emotional adaptation via indirectly via basic psychological needs satisfaction. Therefore, psychological needs satisfaction could serve as a mechanism to explain why left-behind children are more likely to experience depression and loneliness in response to parents’ absence, which supported Ben-Zur’s (2003) conclusions. Warm, close parent–child relationships could promote child development and enhance their well-being. Self-determination theory posited that basic psychological needs satisfaction mediated the effects of environmental factors on psychological health (Deci and Ryan, 2000; Ryan and Deci, 2000). Similarly, Deci and Ryan (2011) claimed that environmental factors that satisfy basic psychological needs promote development and mental health; otherwise, individuals pursue new activities to satisfy these needs. This is encouraging, as it is inconsistent with our initial assumption that migrant parents became “bystanders” in left-behind children’s lives (Zhao et al., 2013b). Even though parents migrate to urban areas for employment, they remain the most important attachment figures and sources of emotional support for left-behind children in middle childhood by using the telephone, messages, and QQ and WeChat communication applications and returning home when possible. The important roles of both external resources (e.g., parent–child cohesion) and individual’s inherent resources (e.g., basic psychological needs satisfaction) in left-behind children’s development should be considered when examining their psychological development, to reduce the adverse effect of being left behind.

In addition, the findings demonstrated both similarities and differences in the experiences of emotional adaptation between boys and girls. The results indicated that the likelihood that left-behind boys would experience depression and loneliness decreased as the closeness of their parent–child relationships increased. Moreover, the quality of parent–child relationships protected left-behind boys’ emotional adaptation to a greater extent, relative to that of girls, and they benefited more from parental closeness, warmth, support, and love than girls did. This was consistent with Tian et al.’s (2014) study, which showed that girls were affected less strongly by parent–child relationships relative to boys. Meanwhile, basic psychological needs satisfaction in girls exerted a significantly stronger negative predictive effect on emotional adaptation, relative to that observed in boys, which could be explained by differences in sociocultural gender expectations between boys and girls. Witt and Wood (2010) asserted that girls are required to be friendly, thoughtful, and sympathetic, while boys are expected to be independent, brave, and competent. Social expectations of men are achievement oriented, while those of women are relationship oriented; therefore, boys are likely to experience greater pressure relative to girls. In contrast, girls tend to have delicate feelings and pay more attention to emotional needs (Stroud et al., 2002). Therefore, left-behind girls are more capable of maintaining high parent–child cohesion levels with their parents (Zhao et al., 2013a) and they may find it easier to obtain basic psychological needs satisfaction, particularly with respect to relatedness. This could enhance the protective effect of girls’ basic psychological needs satisfaction on emotional adaptation. In summary, our findings provide empirical evidence indicating that gender moderated the influence of parent–child cohesion on emotional adaptation in left-behind children, which increases our understanding as to why left-behind children of different genders exposed to similar parental migration stressors differ in emotional adaptation.

This study was subject to several limitations, which should be considered when evaluating the findings. First, all data were collected via self-report questionnaires, which could have resulted in self-report bias. In future, data should be collected from multiple informants, such as parents, guardians, and teachers, to increase data accuracy. Second, the cross-sectional design limited our ability to infer causality in the relationships between parent–child cohesion, basic psychological needs satisfaction, and emotional adaptation; future prospective studies should be conducted to address this issue. Longitudinal designs would allow further examination of causality in these relationships in Chinese left-behind children. Third, the effect of parent–child cohesion on emotional adaptation was examined in left-behind children, but the specific roles of mother– and father–child cohesion were not discussed. Some studies have shown that father– and mother–child cohesion could exert different protective effects on negative emotional outcomes in left-behind children (Vieno et al., 2009; Zhao et al., 2015; Zhao J. X. et al., 2016), which should be examined further in future studies.

Despite these limitations, the study had several strengths and provided a unique contribution to the enhancement of understanding of the potential indirect effects of parent–child cohesion on emotional adaptation via basic psychological needs satisfaction. In addition, it provides preliminary information regarding parent–child cohesion, which could promote positive emotional outcomes in left-behind children. Left-behind children in middle childhood were recruited as participants. Relative to children who lived with their parents, left-behind children were placed in disadvantageous situations as a consequence of being left behind and the long-term absence of parents in everyday activities, which weakened family bonds and created challenges for the children. This study could aid the interpretation of fundamental theoretical issues in developmental psychology, such as the relationship between family environment and children’s psychological development. In addition, the study suggests that the protective effects of parent–child cohesion and basic psychological needs satisfaction in left-behind children could provide empirical support for the application of ecological systems and self-determination theories in left-behind children. Further, we performed a comprehensive analysis of the model of the indirect effect of parent–child cohesion on emotional adaptation via basic psychological needs satisfaction in left-behind children. To our knowledge, this study was one of the first to examine the potential mechanisms underlying parent–child cohesion’s effects on emotional adaptation in Chinese left-behind children. It suggests that satisfying their basic needs for intimacy, autonomy, and competence to the greatest possible extent by encouraging communication and preserving close connections between parents and children could be an effective strategy for improving emotional adaptation in left-behind children. Further, researchers should pay attention to the difference in the effects of parent–child cohesion on emotional adaptation in boys and girls. The similarities and differences analyses enriched our perspective on parent–child cohesion’s impact on left-behind children’s emotional adaptation in both genders, which could help families to develop gender-based, parent–child relationships.

## Ethics Statement

The study was conducted according to the Declaration of Helsinki and was approved by the institutional Review Board at Southwest University.

## Author Contributions

JS and LZ conducted the analyses, interpreted the data, and drafted the manuscript. YR constructed the figures. LX and QZ provided critical revisions. All authors approved the final version of the manuscript for submission.

## Conflict of Interest Statement

The authors declare that the research was conducted in the absence of any commercial or financial relationships that could be construed as a potential conflict of interest.

## References

[B1] All-China Women’s Federation (2013). *China’s Rural Left-Behind Children, Rural and Urban Migrant Children Research Report.* Avaiable at: http://www.hbsfl.gov.cn/diaoyansikao/2013-12-02/577.html

[B2] Asher S. R. Hymel S. Renshaw P. D. (1984). Loneliness in children. *Child Dev.* 55 1456–1464. 10.2307/1130015

[B3] Bean R. A. Barber B. K. Crane D. R. (2006). Parental support, behavioral control, and psychological control among African American youth the relationships to academic grades, delinquency, and depression. *J. Fam. Issues* 27 1335–1355. 10.1177/0192513X06289649

[B4] Ben-Zur H. (2003). Happy adolescents: the link between subjective well-being, internal resources, and parental factors. *J. Youth Adolesc.* 32 67–79. 10.1023/A:1021864432505

[B5] Birleson P. (1981). The validity of depressive disorder in childhood and the development of a self-rating scale: a research report. *J. Child Psychol. Psychiatry* 22 73–88. 10.1111/j.1469-7610.1981.tb00533.x 7451588

[B6] Chen L. Zhang L. J. Shen J. (2009). The effect of parent-child relationship on left-behind children’s subjective well-being in rural China. *Chin. J. Spec. Educ.* 105 8–12.

[B7] Cheng J. Sun Y. H. (2015). Depression and anxiety among left-behind children in China: a systematic review. *Child Care Health Dev.* 41 515–523. 10.1111/cch.12221 25495395

[B8] Cheng P. Da C. Cao F. Li P. Feng D. Jiang C. (2010). A comparative study on psychological abuse and neglect and emotional and behavioral problems of left-behind children and non-left-behind children in rural areas. *Chin. J. Clin. Psychol.* 18 250–253.

[B9] Cicchetti D. Rogosch F. A. (2012). Neuroendocrine regulation and emotional adaptation in the context of child maltreatment. *Monogr. Soc. Res. Child Dev.* 77 87–95. 10.1111/j.1467-8624.2009.01393.x 20331666 PMC2846099

[B10] Collins W. A. Russell G. (1991). Mother-child and father-child relationships in middle childhood and adolescence: a developmental analysis. *Dev. Rev.* 11 99–136. 10.1016/0273-2297(91)90004-8

[B11] Deci E. L. Ryan R. M. (2000). The “what” and “why” of goal pursuits: human needs the self-determination of behavior. *Psychol. Inq.* 11 227–268. 10.1080/08870440902783628 20204932

[B12] Deci E. L. Ryan R. M. (2008). Facilitating optimal motivation and psychological well-being across life’s domains. *Can. Psychol.* 49 14–23. 10.1037/0708-5591.49.1.14

[B13] Deci E. L. Ryan R. M. (2011). Levels of analysis, regnant causes of behavior and well-being: the role of psychological needs. *Psychol. Inq.* 22 17–22. 10.1080/1047840X.2011.545978

[B14] Fan X. H. (2011). On the comparison of emotional adaptation between left-at-home rural children of various types and normal children. *Chin. J. Spec. Educ.* 128 71–77.

[B15] Fan X. H. Fang X. Y. Lin D. H. Zhu D. (2013). Family atmosphere and psychological adjustment among left-behind children: the mediating of social support. *Hunan Soc. Sci.* 25 128–131.

[B16] Fan X. H. Fang X. Y. Liu Q. X. Liu Y. (2009). A social adaptation comparison of migrant children, rear children, and ordinary children. *J. Beijing Norm. Univ.* 215 33–40.

[B17] Fang S. Y. Sang Q. S. Gu Y. T. (2012). The research on the relationship among peer support, basic psychological needs social adaptation of high school students. *Sci. Soc. Psychol.* 27 57–62.

[B18] Feldman R. S. (2006). *Development Across the Life Span.* Hoboken, NJ: Pearson Education, Inc.

[B19] Formoso D. Gonzales N. A. Aiken L. S. (2000). Family conflict and children’s internalizing and externalizing behavior: protective factors. *Am. J. Community Psychol.* 28 175–199. 10.1023/A:1005135217449 10836090

[B20] Fuligni A. J. Zhang W. (2004). Attitudes toward family obligation among adolescents in contemporary urban and rural China. *Child Dev.* 75 180–192. 10.1111/j.1467-8624.2004.00662.x 15015683

[B21] Guo H. Y. Zhu W. L. Zhu Q. Zhu M. L. Zuo P. Y. Lin D. H. (2014). Parent-child communication and perceived well-being: the mediating effects of basic psychological needs satisfaction among rural children in China. *Psychol. Dev. Educ.* 30 129–136.

[B22] Guo J. Ren X. Wang X. Qu Z. Zhou Q. Ran C. (2015). Depression among migrant and left-behind children in china in relation to the quality of parent-child and teacher-child relationships. *PLoS One* 10:e0145606. 10.1371/journal.pone.0145606 26719895 PMC4699918

[B23] Hao Z. Cui L. J. (2007). A study on the influence of self-esteem and locus of control on left-at-home children’s social adaptation. *Psychol. Sci.* 30 1199–1201.

[B24] Kliewer W. Murrelle L. Prom E. Ramirez M. Obando P. Sandi L. (2006). Violence exposure and drug use in Central American youth: family cohesion and parental monitoring as protective factors. *J. Res. Adolesc.* 16 455–478. 10.1111/j.1532-7795.2006.00502.x

[B25] Lamborn S. D. Felbab A. J. (2003). Applying ethnic equivalence and cultural values models to African-American teens’ perceptions of parents. *J. Adolesc.* 26 601–618. 10.1016/S0140-1971(03)00059-9 12972272

[B26] Levin K. A. Currie C. (2010). Family structure, mother-child communication, father-child communication, and adolescent life satisfaction: a cross-sectional multilevel analysis. *Health Educ.* 110 152–168. 10.1108/09654281011038831

[B27] Li Q. F. (2002). Investigation report on the impact of labors in countryside work in the cities upon their children’s development in three provinces. *Shanghai Res. Educ.* 22 25–28.

[B28] Li X. M. Luo J. Gao W. B. Yuan J. (2009). Research on negative emotions, coping style, self-esteem and interpersonal relationship of college students with left-behind experience. *Chin. J. Clin. Psychol.* 17 620–622.

[B29] Lin L. L. He H. B. (2012). The impact of the deficient parent-child relationship on the emotion and morality development of left-behind children and its countermeasure. *J. Hunan First Norm. Coll.* 12 31–35.

[B30] Liu X. Fan X. H. Shen J. L. (2007). Relationship between social support and problem behaviors of the left-home-kids in junior middle school. *Psychol. Dev. Educ.* 22 98–102.

[B31] Liu X. H. Wang X. J. Yang Y. Y. Ha L. N. Li Q. L. Dai X. Y. (2012). Comparison of mental health status between left-behind children under different guardianship and non-left-behind children. *Chin. Gen. Pract.* 15 1507–1510.

[B32] Liu Z. Li X. Ge X. (2009). Left too early: the effects of age at separation from parents on Chinese rural children’s symptoms of anxiety and depression. *Am. J. Public Health* 99 2049–2054. 10.2105/AJPH.2008.150474 19762669 PMC2759782

[B33] Luthar S. S. Cicchetti D. Becker B. (2000). The construct of resilience: a critical evaluation and guidelines for future work. *Child Dev.* 71 543–562. 10.1111/1467-8624.00164 10953923 PMC1885202

[B34] Ministry of Civil Affairs (2016). *The Mystery of the Sharp Drop of Left-Behind Children: From 61.02 Million to 9.02 Million.* Available at: http://www.mca.gov.cn/article/gk/jd/shsw/201611/20161115002462.shtml

[B35] Olson D. H. Sprenkle D. H. Russell C. S. (1979). Circumplex model of marital and family systems: I. Cohesion and adaptability dimensions, family types, and clinical applications. *Fam. Process* 18 3–28. 10.1111/j.1545-5300.1979.00003.x 437067

[B36] Pittman K. J. Irby M. Tolman J. Yohalem N. Ferber T. (2011). *Preventing Problems, Promoting Development, Encouraging Engagement.* Washington, DC: Forum for Youth Investment.

[B37] Ryan R. M. Deci E. L. (2000). Self-determination theory and the facilitation of intrinsic motivation, social development, and well-being. *Am. Psychol.* 55 68–78. 10.1037/0003-066X.55.1.68 11392867

[B38] Shen J. L. Liu X. Zhao J. X. Shi B. G. (2015). The psychological development of Chinese left-behind children and migrant children in urbanization process. *Psychol. Dev. Educ.* 31 108–116.

[B39] Steinberg L. D. (1990). “Interdependence in the family: autonomy, conflict, harmony in the parent-adolescent relationship,” in *At the Threshold: The Developing Adolescent* eds Feldman S. S. Elliott G. R. (Cambridge, MA: Harvard University Press).

[B40] Steinberg L. D. (2007). *Adolescence* 8th Edn. New York, NY: McGraw-Hill.

[B41] Stroud L. R. Salovey P. Epel E. S. (2002). Sex differences in stress responses: social rejection versus achievement stress. *Biol. Psychiatry* 52 318–327. 10.1016/S0006-3223(02)01333-1 12208639

[B42] Su L. Y. Wang K. Zhu Y. Luo X. R. Yang Z. W. (2003). Norm of the depression self-rating scale for children in Chinese urban children. *Chin. Ment. Health J.* 17 547–549.

[B43] Su S. Li X. Lin D. Xu X. Zhu M. (2013). Psychological adjustment among left-behind children in rural China: the role of parental migration and parent–child communication. *Child Care Health Dev.* 39 162–170. 10.1111/j.1365-2214.2012.01400.x 22708901

[B44] Tian L. M. Chen G. H. Wang S. Q. Liu H. J. Zhang W. X. (2012). Effects of parental support and friendship support on loneliness and depression during early and middle adolescence. *Acta Psychol. Sin.* 44 944–956. 10.3724/SP.J.1041.2012.00944

[B45] Tian L. M. Zhang W. X. Chen G. H. (2014). Effects of parental support, friendship quality on loneliness and depression: to test an indirect effect model. *Acta Psychol. Sin.* 46 238–251. 10.3724/SP.J.1041.2014.00238

[B46] Valtolina G. G. Colombo C. (2012). Psychological well-being, family relations, and developmental issues of children left behind. *Psychol. Rep.* 111 905–928. 10.2466/21.10.17.PR0.111.6.905-928 23402056

[B47] Vieno A. Nation M. Pastore M. Santinello M. (2009). Parenting and antisocial behavior: a model of the relationship between adolescent self-disclosure, parental closeness, parental control, and adolescent antisocial behavior. *Dev. Psychol.* 45 1509–1519. 10.1037/a0016929 19899910

[B48] Von Korff L. Grotevant H. D. (2011). Contact in adoption and adoptive identity formation: the mediating role of family conversation. *J. Fam. Psychol.* 25 393–401. 10.1037/a0023388 21517175 PMC3465677

[B49] Wang L. Mesman J. (2015). Child development in the face of rural-to-urban migration in China: a meta-analytic review. *Perspect. Psychol. Sci.* 10 813–831. 10.1177/1745691615600145 26581737

[B50] Wang M. P. Zhang W. X. (2007). A research on parent-adolescent conflict and cohesion. *Psychol. Sci.* 30 1196–1198.

[B51] Wei M. Shaffer P. A. Young S. K. Zakalik R. A. (2005). Adult attachment, shame, depression, and loneliness: the mediation role of basic psychological needs satisfaction. *J. Couns. Psychol.* 52 591–601. 10.1037/0022-0167.52.4.591

[B52] Wen M. Lin D. (2012). Child development in rural China: children left behind by their migrant parents and children of non-migrant families. *Child Dev.* 83 120–136. 10.1111/j.1467-8624.2011.01698.x 22181046

[B53] Wen Z. L. Hau K. T. Marsh H. W. (2004). Structural equation model testing: cutoff criteria for goodness of fit indices and chi-square test. *Acta Psychol. Sin.* 36 186–194.

[B54] Witt M. G. Wood W. (2010). Self-regulation of gendered behavior in everyday life. *Sex Roles* 62 635–646. 10.1097/ANS.0b013e3181eb4215 20693831

[B55] Xiao S. R. Xu G. X. (2009). Gender differences: influence of family environment on personality traits among adolescents. *Psychol. Explor.* 29 71–75.

[B56] Yang Q. S. Zhou L. Hu Y. Q. Zhu C. Y. Sun H. L. (2014). Influence of parent-adolescent communication on behavioral problems of left-behind children. *Chin. J. Clin. Psychol.* 22 1118–1120.

[B57] Yu C. F. Zhang W. Zeng Y. Y. Ye T. Hu J. P. Li D. L. (2012). Gratitude, basic psychological needs problematic Internet use in adolescence. *Psychol. Dev. Educ.* 28 83–90.

[B58] Zeng S. C. Li Q. W. (2003). A review of research on the development of children’s psychological resilience. *Psychol. Sci.* 26 1091–1094.

[B59] Zhang W. X. Fuligni A. J. (2006). Authority, autonomy, and family relationships among adolescents in urban and rural China. *J. Res. Adolesc.* 16 527–537. 10.1111/j.1532-7795.2006.00506.x

[B60] Zhang W. X. Wang M. P. Fuligni A. J. (2006). Expectations for autonomy, beliefs about parental authority, and parent-adolescent conflict and cohesion. *Acta Psychol. Sin.* 38 868–876. 9681270

[B61] Zhao J. X. Liu X. Li Y. (2013a). Daily hassles and rural left-behind children’s delinquent behavior: the role of parental cohesion. *Psychol. Dev. Educ.* 28 400–406.

[B62] Zhao J. X. Liu X. Wang M. F. (2015). Parent-child cohesion, friend companionship and left-behind children’s emotional adaptation in rural China. *Child Abuse Negl.* 48 190–199. 10.1016/j.chiabu.2015.07.005 26190190

[B63] Zhao J. X. Liu X. Zhang W. X. (2013b). Peer rejection, peer acceptance and psychological adjustment of left-behind children: the roles of parental cohesion and children’s cultural beliefs about adversity. *Acta Psychol. Sin.* 45 797–810. 10.3724/SP.J.1041.2013.00797

[B64] Zhao J. X. Yang P. Ma J. L. Huang C. C. (2016). Perceived discrimination and positive/negative emotion of left-behind children: the protective role of parent-child cohesion. *Psychol. Dev. Educ.* 32 369–376.

[B65] Zhao K. Yang L. H. Lai Y. Yang Y. Q. Yang M. H. (2016). Effect of perceived social support on well-being among middle school students: mediating role of social adaptation and basic psychological needs. *Chin. J. Sch. Health* 37 1043–1045.

[B66] Zhao X. Chen J. Chen M. C. Lv X. L. Jiang Y. H. Sun Y. H. (2014). Left-behind children in rural China experience higher levels of anxiety and poorer living conditions. *Acta Paediatr.* 103 665–670. 10.1111/apa.12602 24527673

